# Evolution and immunopathology of chikungunya virus informs therapeutic development

**DOI:** 10.1242/dmm.049804

**Published:** 2023-04-04

**Authors:** Filipa Henderson Sousa, Amalina Ghaisani Komarudin, Fern Findlay-Greene, Anom Bowolaksono, R. Tedjo Sasmono, Craig Stevens, Peter G. Barlow

**Affiliations:** ^1^ School of Applied Sciences, Edinburgh Napier University, Sighthill Campus, Edinburgh EH11 4BN, UK; ^2^ Centre for Discovery Brain Sciences and UK Dementia Research Institute, The University of Edinburgh, Edinburgh EH16 4SB, UK; ^3^ Eijkman Research Center for Molecular Biology, National Research and Innovation Agency, Cibinong Science Center, Cibinong, Kabupaten Bogor 16911, Indonesia; ^4^ Cellular and Molecular Mechanisms in Biological System (CEMBIOS) Research Group, Department of Biology, Faculty of Mathematics and Natural Sciences, Universitas Indonesia, Depok 16424, Indonesia

**Keywords:** Chikungunya virus, Chikungunya pathogenesis, Antiviral compounds, Vaccines, Monoclonal antibodies, Immunomodulatory drugs

## Abstract

Chikungunya virus (CHIKV), a mosquito-borne alphavirus, is an emerging global threat identified in more than 60 countries across continents. The risk of CHIKV transmission is rising due to increased global interactions, year-round presence of mosquito vectors, and the ability of CHIKV to produce high host viral loads and undergo mutation. Although CHIKV disease is rarely fatal, it can progress to a chronic stage, during which patients experience severe debilitating arthritis that can last from several weeks to months or years. At present, there are no licensed vaccines or antiviral drugs for CHIKV disease, and treatment is primarily symptomatic. This Review provides an overview of CHIKV pathogenesis and explores the available therapeutic options and the most recent advances in novel therapeutic strategies against CHIKV infections.

## Teaser

**Summary:** Although chikungunya virus (CHIKV) cases are rising globally, therapeutic options remain extremely limited. Here, we provide an overview of CHIKV pathogenesis and explore recent advances in strategies to prevent and treat infections.

## Introduction

Chikungunya virus (CHIKV) disease causes severe, incapacitating joint pain, which may persist for several months or years, resulting in serious economic and social impact for affected communities (Mohan et al., 2010). CHIKV is an arthropod-borne virus (see Glossary, Box 1) that belongs to the *Togaviridae* family (genus *Alphavirus*) (Box 1, Box 2) and is transmitted by two species of mosquitoes, *Aedes albopictus* and *Aedes aegypti* (Dubrulle et al., 2009; Nuckols et al., 2013; Díaz-González et al., 2015). CHIKV was first recognised as a human pathogen in 1952, when it was isolated from human serum during an epidemic in Tanzania (Robinson, 1955). The origin of the name chikungunya comes from a Makonde word that translates as “that which bends up”, a reference to the contorted posture exhibited in infected patients with severe joint pain characteristic of the disease (Robinson, 1955).

CHIKV disease in humans is typically marked by two phases, an acute phase and a chronic phase. Symptoms of CHIKV infection start abruptly, normally presenting with a high fever (>38.9°C) that can last from several days to up to 2 weeks. The majority of infected patients develop polyarthralgia (Box 1) after the onset of fever, but other common symptoms can include rash, myalgia (Box 1) and headaches (Burt et al., 2017). After the acute phase of the illness, some patients develop long-term symptoms, known as the chronic phase, that can last from several weeks (Chopra et al., 2012) to months or years (Chang et al., 2018; Paixão et al., 2018). Studies vary widely in terms of the percentage of patients that experience the chronic disease and the disease longevity. Further research is warranted to better assess the burden of chronic disease upon inflicted populations with CHIKV epidemics.

The long-term sequelae include arthralgia (Box 1), joint stiffness, swelling and tendonitis/tenosynovitis (Box 1), as well as alopecia and depression (Hawman et al., 2013; van Aalst et al., 2017). CHIKV-induced arthritis resembles rheumatoid arthritis (RA), but, unlike RA, there is no evidence that CHIKV-associated arthropathies are caused by autoimmunity. Although the case fatality rate for CHIKV disease has been estimated to be 0.009% [Pan American Health Organization/World Health Organization Annual Arbovirus Bulletin, 2021], a meta-analysis study by Rodríguez-Morales et al. (2016) predicted that 25% of CHIKV infections would lead to chronic inflammatory rheumatism and 14% of patients would develop chronic arthritis. However, the estimates of the rate of progression to chronic arthritis post-CHIKV infection vary widely due to the criteria used and the populations examined. Therefore, a more recent systematic review suggests that, on average, 42.5% of patients experience chronic arthritis post-CHIKV infection (Puntasecca et al., 2021). The population risk groups identified as being more likely to develop severe disease include infants, the elderly and immunocompromised individuals (Sebastian et al., 2009; Lang et al., 2017).

In the past 20 years, CHIKV has re-emerged as a major threat to public health with worldwide distribution and has been associated with frequent regional outbreaks (Box 3). In the most recent outbreak in 2013 starting in the Caribbean island of Saint Martin, CHIKV spread to 22 countries in the Caribbean and Central and South America, resulting in hundreds of thousands of infections (Van Bortel et al., 2014; Kautz et al., 2015). The rapid spread of CHIKV worldwide is a clear indicator that vector control (Box 1) strategies, which are currently the only method available to protect populations from CHIKV and other vector-borne viruses, are insufficient to contain arboviral diseases. To date, there are no licensed vaccines or preventative approaches for the targeted treatment of CHIKV. Although several vaccine strategies are being pursued, with some in various stages of clinical trials, these vaccines are still several years away from being licensed and available to the public. Currently, the best protection against CHIKV disease is preventing infection by avoiding mosquito bites. Furthermore, with no approved antiviral therapy, the existing treatment for patients with CHIKV disease is based on supportive care and consists of the administration of analgesics, antipyretics and non-steroid anti-inflammatory drugs (NSAIDs). Effective therapeutic and preventative approaches for CHIKV infection are urgently required.

In this Review, we provide a current perspective on CHIKV pathogenesis, as well as novel therapeutic strategies used to prevent, treat and manage CHIKV disease, including vaccines, monoclonal antibodies, antiviral compounds and immunomodulatory drugs.

**Box 1. Glossary**

**Arthralgia:** pain in a joint.

**Arthropod-borne virus**: also known as an arbovirus, a general term used to describe a group of viruses that spread to humans by the bite of arthropods (insects), such as mosquitoes or ticks. Chikungunya virus (CHIKV) is transmitted to humans by the bite of infected mosquitoes.

**Autochthonous transmission:** spread of a disease from one individual to another individual in the same location.

**Myalgia:** muscle pain.

**Neutrophil extracellular trap (NET)**: a network of extracellular fibres, mainly composed of DNA associated with host defence peptides, which can engulf and kill pathogens.

**Osteoclast:** large, multinucleated cell responsible for bone resorption (bone destruction and release of minerals into blood). Osteoclasts are derived from precursors in the myeloid/monocyte lineage that circulate in the blood.

**Polyarthralgia:** pain in several joints.

**Structure–activity relationship:** the relationship between the chemical structure of a compound and its biological effect.

**Tendonitis:** inflammation of a tendon.

**Tenosynovitis:** inflammation of the synovial fluid that surrounds a tendon; associated with tendonitis.

***Togaviridae* family:** a family of enveloped viruses with single-stranded positive-sense RNA genomes of 10-12 kb. *Alphavirus* is a genus within the *Togaviridae* family and includes a large number of viruses transmitted by arthropods, typically mosquitoes. Well-studied members of this genus include CHIKV, Sindbis virus, Semliki Forest virus, Venezuelan equine encephalitis virus and Ross River virus.

**Vector control:** limiting the transmission of a virus by reducing or eliminating human contact with mosquitos, through the use of chemical and non-chemical based tools (reviewed in Wilson et al., 2020).

**Box 2. CHIKV structure**

CHIKV is an enveloped positive-sense RNA alphavirus of ∼60-70 nm in diameter. The viral genome consists of single-stranded, linear RNA that is 11.8 kb in size, encompassing two open reading frames (ORFs), encoding two polyproteins. ORF1 encodes for non-structural proteins (nsPs; nsP1, helicase nsP2, nsP3, polymerase nsP4) and ORF2 encodes for structural proteins [capsid (C) protein, envelope proteins (E1, E2, E3), 6K] (Voss et al., 2010; Ahola and Merits, 2016).

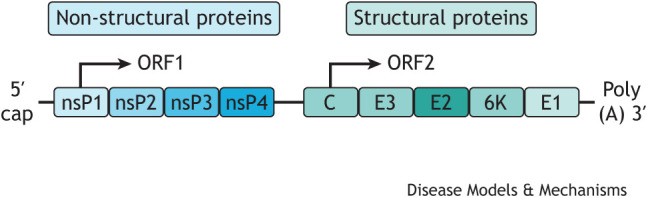

The nsPs are important for viral replication; however, none of these proteins are packaged in the final virion (Powers, 2017). The structural surface glycoproteins E1 and E2 assemble into spikes on the virion surface – each spike consisting of a trimer of E2-E1 heterodimers – and are the major viral epitopes responsible for the attachment and entry into the host cell.

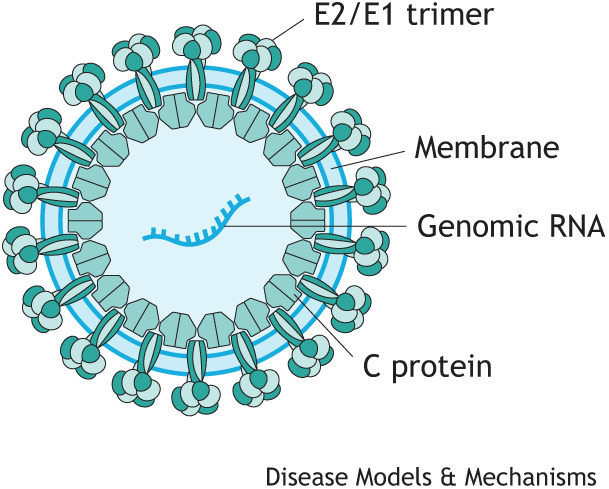

The E1 glycoprotein is necessary for membrane fusion and the E2 protein is responsible for receptor binding (Voss et al., 2010). The E3 glycoprotein serves as a signal sequence for the translocation of E3-E2-6K-E1 polyprotein into the endoplasmic reticulum, where it undergoes complete cleavage into individual proteins important for virus maturation and spike assembly (Snyder and Mukhopadhyay, 2012). The C protein associates with the genomic RNA to form a nucleocapsid that is coated with surface proteins E1 and E2.

**Box 3. Evolution and spread of CHIKV**

Phylogenetic analysis of CHIKV has identified four distinct lineages with different genotypes corresponding to their geographic region, including Asian (sub-lineage Asian/American), East/Central/South African (ECSA), West African and Indian Ocean lineage (IOL) genotypes (Wahid et al., 2017). In 2004, CHIKV re-emerged in coastal Kenya and spread throughout the western Indian Ocean islands (Chretien et al., 2007). This led to the most recent major epidemic of CHIKV that occurred between 2005 and 2006 and affected several countries located in the Indian Ocean. On La Réunion island, one-third of the population was infected with CHIKV (Vazeille et al., 2007; Staples et al., 2009). The CHIKV isolated during this epidemic represented a novel ECSA genotype with a mutation in the E1 envelope glycoprotein (E1-A226V), which was subsequently described as the IOL (Sahadeo et al., 2015). As CHIKV glycoproteins play an important role in viral transmission, emergence and spread, the E1-A226V mutation increased infectivity and transmission by *Aedes albopictus* mosquitoes, with less preferential transmission by *Aedes aegypti* (Tsetsarkin et al., 2007). Other mutations within the IOL have been identified on CHIKV envelope glycoprotein E2 (e.g. L210Q, K252Q), which further increase viral fitness in *A. albopictus* (Tsetsarkin et al., 2014). The mosquito *A. albopictus* is more abundant and widely distributed than *A. aegypti* in southern Europe and America, and several CHIKV outbreaks and autochthonous transmission (Box 1) have been reported recently in these regions, including Italy (Rezza et al., 2007), France (Roiz et al., 2015), Spain, Caribbean islands (Van Bortel et al., 2014), Argentina, Mexico (Kautz et al., 2015) and USA (Madariaga et al., 2016). CHIKV isolates originating from the 2013 Saint Martin outbreak, which spread throughout the Caribbean and into Central and South America, also form a novel American sublineage within the Asian lineage (Asian/American) (Van Bortel et al., 2014; Kautz et al., 2015). The continuous evolution of CHIKV glycoproteins increases the virus’ fitness in a more widely distributed vector, which expands its geographical impact. Owing to globalisation and the year-round presence of mosquito vectors, especially in highly populated urban areas (Kraemer et al., 2015), there is a high risk that the virus will become endemic in several different regions of the world.

## CHIKV infection and host immune responses

**Fig. 1.**
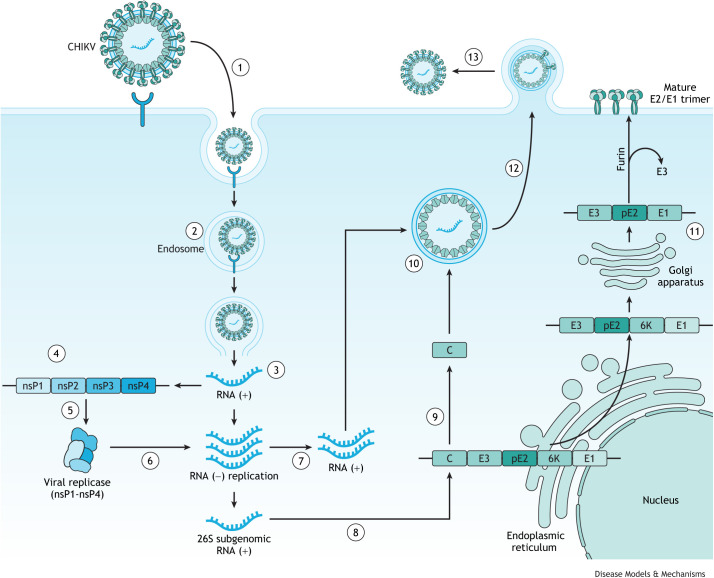
**CHIKV life cycle.** (1) CHIKV enters the host cells through receptor-mediated endocytosis. The viral protein E2 interacts with specific host surface receptors. A number of receptors [for example, prohibitin (Wintachai et al., 2012), MXRA8 (Zhang et al., 2018), PS receptors (Moller-Tank et al., 2013) and GAG (Silva et al., 2014)] have been implicated in this process. (2) Once in the endosome, the acidic pH triggers conformational changes in the viral envelope, exposing the E1 peptide and leading to its fusion with the endosomal membrane (Voss et al., 2010). (3) This allows for the cytoplasmic delivery of the nucleocapsid and the release of the viral RNA genome. (4) The viral genome is translated to the non-structural polyprotein nsP1- nsP2-nsP3-nsP4. (5) The viral protease nsP2 then cleaves the polyprotein into the individual nsPs – nsP1, nsP2, nsP3 and nsP4 – that then form the viral replicase complex. (6,7) The viral replicase is responsible for the synthesis of the negative-strand RNA that will be a template for both new positive-strand RNA and the sub-genomic RNA (26S RNA) (van der Heijden and Bol, 2002). (8) The 26S RNA drives the expression of the structural polyprotein C-pE2-6K-E1 in the endoplasmic reticulum. (9,10) The capsid protein (C) dissociates from the polyprotein by self-cleavage activity and binds to the newly synthesised viral RNA, forming the nucleocapsid core in the cytoplasm. (11) In the meantime, E2 and E1 associate in the Golgi apparatus and are exported to the cell membrane, where pE2 is cleaved by the host protease furin into E2 and E3. E3, which stabilises the E2/E1 trimer, then dissociates from the trimer when it reaches the cell membrane. (12) The already-formed nucleocapsid migrates to the host cell membrane region rich in E2/E1 trimers that bind the virion membrane. (13) The mature virions are released by the budding process from the infected cells (Yap et al., 2017). CHIKV, chikungunya virus; GAG, glycosaminoglycan; MXRA8, matrix remodelling-associated protein 8; nsP, non-structural protein; PS receptor, phosphatidylserine-mediated entry-enhancing receptor.

**Fig. 2.**
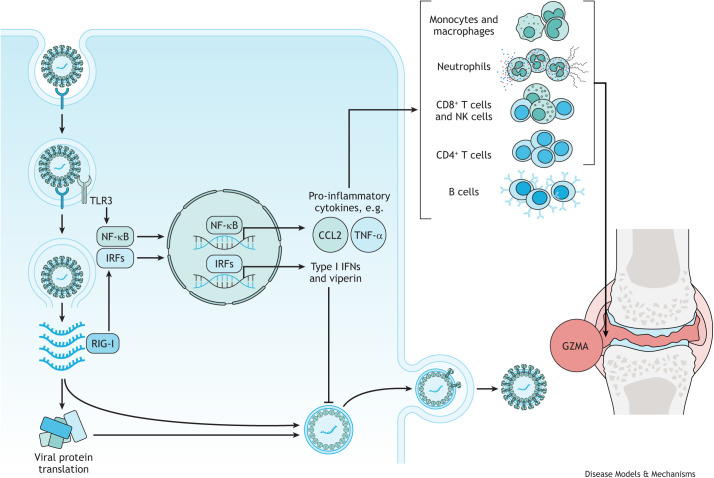
**CHIKV infection activates host immune responses, leading to joint/muscle inflammation.** CHIKV is detected by TLR3, TLR7 and TLR8, as well as by RIG-I-like receptors, which activate transcription factors, NF-κB and IRFs. IRFs stimulate a strong antiviral type I IFN response through the transcription of IFN-stimulated genes that encode antiviral proteins, such as viperin, IFN-α/β and OAS (Teng et al., 2012; Priya et al., 2014; Onomoto et al., 2021). NF-κB activates a pro-inflammatory response by stimulating the transcription of many pro-inflammatory cytokines and chemokines, including TNF-α and CCL2 (Teng et al., 2015; Onomoto et al., 2021). The chemokine CCL2 is responsible for the recruitment of monocytes/macrophages to the site of infection, while TNF-α has been linked to the recruitment of cytolytic lymphocytes, including NK cells and CD8^+^ T cells that secrete the pro-inflammatory granule GZMA, which has demonstrated a prominent role in driving arthritic inflammation. Studies have also shown that antibody-producing B cells are involved in CHIKV clearance and control. CD4^+^ T cells were shown to be activated during the chronic phase of CHIKV infection and play a role in pathogenesis of CHIKV-induced joint inflammation. CD8^+^ T cells, neutrophils and monocytes/macrophages can also accumulate in the joints, with monocytes and macrophages even differentiating into osteoclasts that can lead to damage in the joint. CCL2, chemokine ligand 2; CHIKV, chikungunya virus; IFN, interferon; IRF, interferon regulatory factor; GZMA, granzyme A; NF-κB, nuclear factor kappa-light-chain-enhancer of activated B cells; NF-κB, nuclear factor kappa B; NK, natural killer; OAS, 2'-5'-oligoadenylate synthetase 1; RIG-I, retinoic acid-inducible gene I; TLR, Toll-like receptor; TNF-α, tumour necrosis factor alpha.

CHIKV and other arboviruses infect the mosquito midgut, following the ingestion of viraemic blood, then replicate and disseminate to the salivary glands, being transmitted via saliva when the mosquito bites. Following the mosquito bite, CHIKV replicates at the site of the inoculation (Fig. 1). The dermal fibroblasts have been shown to constitute the main site of viral amplification (Ekchariyawat et al., 2015; Wichit et al., 2017). The spread of the virus from the skin to other peripheral organs is thought to be via the circulatory system (Fig. 2), as CHIKV was shown to infect human peripheral blood mononuclear cells (PBMCs) *in vitro*, with monocytes primarily infected and B cells and myeloid dendritic cells to a lesser extent infected (Her et al., 2010; Ruiz Silva et al., 2016). CHIKV antigens were also detected *in vivo* in the monocytes of acutely infected patients (Her et al., 2010). In contrast, a previous study by Sourisseau et al. (2007) showed that PBMCs – including monocytes, B cells, T cells and monocyte-derived dendritic cells – were not susceptible to CHIKV infection. These contradictory results indicate that more research is required.

Myalgia and arthralgia are two main symptoms associated with CHIKV reaching the muscles and joints, predominantly attributed to infection of myoblasts, skeletal muscle fibroblasts and synovial fibroblasts, as well as to joint macrophages (Dubrulle et al., 2009; Hoarau et al., 2010; Phuklia et al., 2013; Lohachanakul et al., 2015; Guerrero-Arguero et al., 2020). The ability of CHIKV to affect multiple systems/organs might be due to the widespread expression of receptors, which permits infection of a wide range of different cell types. In mammalian cells, suggested receptors for CHIKV include prohibitin (Wintachai et al., 2012), matrix remodelling-associated protein 8 (MXRA8) (Zhang et al., 2018), phosphatidylserine-mediated entry-enhancing receptors (PS receptors) (Moller-Tank et al., 2013) and glycosaminoglycans (GAGs) (Silva et al., 2014); however, their precise roles are not well elucidated.

### Acute phase of CHIKV disease

The acute phase of CHIKV disease lasts typically <21 days after the onset of infection. It is divided into two different stages, the viraemic phase (5-10 days), marked by high fever and polyarthralgia/arthritis, myalgia, headaches and skin rashes; and the post-viraemic phase (6-21 days), characterised by a lack of fever, polyarthralgia/arthritis and, to a lesser extent, myalgia, fatigue and anorexia (Thiberville et al., 2013).

During the acute phase of infection, CHIKV RNA is detected by pattern recognition receptors, including Toll-like receptor (TLR)3, TLR7 and TLR8, as well as retinoic acid-inducible gene I (RIG-I)-like receptors, which trigger the production of type I interferons (IFNs) through the activation of the transcription factors IFN regulatory factors (IRFs) and nuclear factor kappa B (NF-κB). Both IRFs and NF-κB induce the transcription of IFN-stimulated genes (ISGs) and pro-inflammatory cytokine genes (Onomoto et al., 2021). Type I ISGs represented 50% of all upregulated genes in an RNA-sequencing (RNA-seq) analysis (Wilson et al., 2017), with IFN-α and IFN-γ, in particular, upregulated in all acutely infected patients during the first week of symptoms (Wauquier et al., 2011). IFNs, and specifically type I IFNs, appear to play essential roles in controlling the severity of CHIKV infection. Of note, results from CHIKV infection of mice deficient in IFN-α/β receptors have suggested that rapid early induction of type I IFNs is required to control virus replication and protect against disease severity (Couderc et al., 2008; Gardner et al., 2012; Rudd et al., 2012). Furthermore, *in vivo* studies have identified fibroblasts as the predominant target cell of CHIKV and the main source of type I IFNs (specifically IFN-β). Type I IFN produced by and acting on fibroblasts appears to be essential for the control and early clearance of CHIKV *in vivo* (Couderc et al., 2008; Schilte et al., 2010).

Many ISGs upregulated during the initial CHIKV infection encode proteins involved in host defence (Teng et al., 2012; Priya et al., 2014). Viperin, an inducible antiviral protein, was elevated in monocytes from CHIKV-infected patients (Teng et al., 2012). Furthermore, in viperin-deficient mice, there was a direct correlation between the lack of viperin expression and higher CHKV replication and joint inflammation (Teng et al., 2012). Hence, the upregulation of viperin could be a potential host strategy to control CHIKV infection.

In addition to a strong antiviral type I response, the acute phase of CHIKV disease is also associated with a potent inflammatory response (Chow et al., 2011; Wauquier et al., 2011; Teng et al., 2015; Wilson et al., 2017). A systematic meta-analysis characterised the circulatory immune mediators in plasma or serum samples from different patient cohorts with acute CHIKV infection and identified upregulation of pro-inflammatory cytokines, including IL-6, IL-8, CCL2, RANTES (also known as CCL5), TNF-α (also known as TNF) and IL-1α, as well as the anti-inflammatory cytokines IL-10 and IL-13, growth factors GM-CSF (also known as CSF2), G-CSF (also known as CSF3) and VEGF, and other mediators IL-4, IL-17, CXCL9, IFN-α and IFN-γ (Teng et al., 2015). The production of pro-inflammatory cytokines and chemokines at the primary sites of infection, mainly joints and muscles, mediates the infiltration of monocytes/macrophages, neutrophils, natural killer (NK) cells and T cells to these infection sites, resulting in tissue inflammation and damage that manifests as arthralgia in patients (Zare et al., 2004; Gardner et al., 2010).

The monocytes/macrophages that are recruited to the sites of inflammation during CHIKV infection have been shown to have antiviral activity against CHIKV (Haist et al., 2017; Fox et al., 2019), and to contribute to the resolution of inflammation and tissue repair (Ikeda et al., 2018). However, the musculoskeletal tissues of CHIKV-infected patients were shown to be greatly infiltrated with monocytes and macrophages (Hoarau et al., 2010), which strongly implicates these cells in CHIKV arthritic immunopathology. Studies in mice in which monocytes/macrophages were depleted (Gardner et al., 2010) or migration was inhibited (Rulli et al., 2011; Chen et al., 2015) suggested that these cells can be pathogenic effectors during CHIKV infection. However, CHIKV infection of mice deficient in CCR2, the receptor for CCL2, a potent monocyte-attracting chemokine, resulted in exacerbation of the arthritic disease, in which the monocytes/macrophages infiltrate was replaced by a severe neutrophil and eosinophil infiltrate (Poo et al., 2014a). Thus, the role of monocytes/macrophages in CHIKV infection is complex, and although their persistent recruitment may contribute to chronic inflammation, initial macrophage recruitment can also prevent excessive pathology.

As mentioned above, neutrophils are also recruited to the site of inflammation during CHIKV infection, where they can produce reactive oxygen species (ROS) and other inflammatory mediators important in fighting infection. Similar to that of macrophages, the role of neutrophils during CHIKV infection is poorly understood. A study using zebrafish demonstrated that CHIKV infection induced a strong type I IFN response and identified neutrophils as the main source of type I IFNs. The depletion of neutrophils in this model led to an increase in disease severity and mortality, which was associated with high viral load, demonstrating the crucial role neutrophils have in fighting CHIKV infection (Palha et al., 2013). Of note, a study by Hiroki et al. (2019) identified neutrophil extracellular traps (NETs; Box 1) as a protective mechanism during CHIKV infection. NETs were shown to directly inhibit CHIKV replication *in vitro*. Furthermore, in IFN receptor-deficient (Ifnar^−/−^) mice, the inhibition of NETs by DNAse treatment resulted in increased susceptibility to CHIKV infection and viral load (Hiroki et al., 2019).

The acute phase is also mediated by CD8^+^ T-cell responses in the early stages of infection, whereas CD4^+^ T-cell responses are present in the later stages of infection (Wauquier et al., 2011). During acute CHIKV infection, activated CD8^+^ T cells were significantly elevated in the peripheral blood of patients, with high expression of cytolytic markers granzyme B and perforin, and the degranulation marker CD107A (also known as LAMP1) (Hoarau et al., 2010; Dias et al., 2018). This suggests that CD8^+^ T cells mediate cytolytic activity against CHIKV-infected cells by releasing the cytolytic granules, perforin and granzyme B. Furthermore, in mice infected with CHIKV, CD4^+^ T cells and CD8^+^ T cells infiltrated inflamed joints (Morrison et al., 2011). Despite this, mice deficient in CD8^+^ T cells still developed joint inflammation (Teo et al., 2013). Interestingly, immunisation with a vaccine that induces virus-specific effector CD8^+^ T cells prior to infection increased the clearance of CHIKV infection in the spleen but not in joint-associated tissues (Davenport et al., 2020). This was also observed in Ross River virus (RRV) infection in mice, in which CD8^+^ T cells contributed to the control of RRV infection in certain tissues but failed to mediate an antiviral response in the joints (Burrack et al., 2015). This suggests that CHIKV establishes and maintains a persistent infection in joint-associated tissues partly by evading CD8^+^ T-cell immunity.

Granzyme A (GZMA), a serine protease granule secreted by cytotoxic lymphocytes, including CD8^+^ T cells, NK cells and natural killer T (NKT) cells, was elevated in the serum of CHIKV-infected patients and correlated with viral loads and disease severity. In a CHIKV mouse model, serum GZMA levels were also elevated and NK cells were attributed as the main source (Schanoski et al., 2019). Previous studies also showed that *Gzma*^−/−^ mice had a pronounced reduction in foot swelling and arthritis compared to wild-type C57BL/6J mice, suggesting a pro-inflammatory role of GZMA in CHIKV infection (Wilson et al., 2017). However, *Gzma*^−/−^ mice have a mixed C57BL/6J and C57BL/6N genetic background and retain the full-length nicotinamide nucleotide transhydrogenase (*Nnt*) gene, whereas in the wild-type C57BL/6J mice the *Nnt* gene is truncated. Hence, the genetic background and the presence of the full-length *Nnt* gene, rather than the loss of GZMA expression, were shown to be responsible for the ameliorated CHIKV arthritis phenotype of *Gzma*^−/−^ mice (Rawle et al., 2022). Nevertheless, this cannot be directly extrapolated to humans, and further studies are warranted to determine whether GZMA could be a potential target for therapy in CHIKV-mediated inflammation.

During the acute phase of infection, CHIKV-specific IgM can be detected by day 4 and CHIKV-specific IgG antibodies can be detected by day 10 (Chua et al., 2017). Numerous studies have established an association between antibody response and viral titres, cytokine levels and disease progression during the acute and chronic phases of infection (Kam et al., 2012; Jain et al., 2017). Patients with high viremia were shown to develop high levels of neutralising IgG3 antibodies. Although these patients underwent a more severe disease progression during the acute viraemic phase, they were able to clear the virus faster and did not develop persistent arthralgia. Conversely, patients that presented low viremia produced IgG3 at a later stage and were shown to develop persistent arthralgia. Therefore, determining the levels of early CHIKV-specific IgG3 could serve as a marker for the identification of patients at risk of developing the severe chronic disease (Kam et al., 2012). In support of this, animal studies have provided evidence of the role of antibodies in CHIKV infection. Lum et al. (2013) showed that CHIKV infection in B-cell knockout mice resulted in viremia that persisted for more than 1 year, with a more severe disease than that of wild-type mice, indicating that antibody-producing cells are directly involved in viral clearance and control (Lum et al., 2013).

Overall, the acute phase of CHIKV infection is characterised by a type I IFN response alongside inflammatory responses that recruit innate and adaptive immune cells. This orchestrated recruitment of leukocytes is important for controlling and resolving infection, but if the fine-tuned balance is disrupted, more severe and/or prolonged symptoms can arise.

### Chronic phase of CHIKV disease

It is estimated that, on average, 42.5% of CHIKV-infected patients will develop arthritis/arthralgia that can persist for several months and even years (Puntasecca et al., 2021). This is known as the chronic phase of CHIKV disease.

The main symptom in chronic CHIKV disease is arthralgia; however, the underlying inflammatory stimuli responsible for chronic CHIKV arthropathy are far from understood. An RNA-seq analysis of CHIKV infection in wild-type mice suggested that chronic arthritic disease represents a prolongation of the acute inflammatory response (Wilson et al., 2017), rather than the activation of new immunopathological inflammatory responses (Suhrbier and Mahalingam, 2009), which continues until the virus material is cleared (Poo et al., 2014b).

During the chronic stage, CHIKV particles have predominantly been cleared from the blood; however, a substantial body of evidence reports that CHIKV RNA and CHIKV-specific proteins can persist in tissues. Hoarau et al. (2010) reported that CHIKV RNA and proteins were detected in synovial macrophages of one patient 1.5 years after infection (Hoarau et al., 2010). CHIKV antigens were also detected in human muscle satellite cells 3 months after acute infection (Ozden et al., 2007). These results are consistent with animal studies that found that CHIKV RNA persisted in muscle fibroblasts in mice (Young et al., 2019) and in macrophages in non-human primates (NHPs) (Labadie et al., 2010). As previously noted, CHIKV-induced arthritis draws several parallels with autoimmune arthritis, although there is very little evidence that viral arthritis leads to autoimmune disease. Rather, it is thought that the persistence of viral antigens could be a contributing factor to the development of chronic CHIKV-induced arthritis. One hypothesis is that double-stranded RNA (dsRNA) intermediates found in infected tissues act as pro-inflammatory pattern-associated molecular patterns that trigger an arthritogenic response (Zare et al., 2004; Hawman et al., 2013; McCarthy and Morrison, 2017).

The chronic phase has consistently been associated with increased levels of circulating IL-6 (Ng et al., 2009; Chow et al., 2011; Noret et al., 2012), as well as GM-CSF, IL-12, IL-17, IL-27, receptor activator of NF-κB ligand (RANKL; also known as TNFSF11) and IL-8 (Hoarau et al., 2010; Chaaitanya et al., 2011; Kelvin et al., 2011; Noret et al., 2012; Gualberto Cavalcanti et al., 2019). The plasma levels of IL-6 in patients with persistent arthralgia were higher than those in convalescent-phase patients (Chow et al., 2011), and this observation was further supported by the findings of Hoarau et al. (2010), showing that IL-6 was specifically expressed in the affected joint during chronic disease. The bone-forming osteoblasts (OBs) express RANKL and osteoprotegerin (OPG; also known as TNFRSF11B), and a high ratio of RANKL/OPG is associated with the formation of osteoclasts (Box 1) from monocytic precursors. CHIKV has been shown to infect OBs, inducing the expression of IL-6 and RANKL and repressing OPG expression (Noret et al., 2012), which would presumably lead to increased osteoclast formation. In addition, CHIKV is known to infect fibroblast-like synoviocytes, which induces the secretion of RANKL, IL-6, IL-8 and CCL2, and leads to the migration and differentiation of monocytes into osteoclasts (Phuklia et al., 2013). The presence of osteoclasts in the joints can lead to damage of the joint structure and contribute to the arthritic-like syndrome, which has been described in RA (Schett, 2007).

T cells play an important role in the development of chronic CHIKV infection. CD8^+^ T cells in patients with chronic disease showed a reduction in activation marker CD69 and in cytolytic activity, demonstrated by the decreased expression of granzyme B, perforin and CD107A, compared to that in patients in the acute phase of the infection (Dias et al., 2018). In the acute phase of the infection, the continuous presentation of CHIKV epitopes by antigen-presenting cells to CD8^+^ T cells leads to the sustained activation of T-cell receptors (Mueller and Ahmed, 2009; Dias et al., 2018). This could cause CD8^+^ T-cell exhaustion, leading to cells shutting down and rendering them unable to eliminate infected cells, which may lead to chronic CHIKV infection with lower levels of functioning CD8^+^ T cells (Mueller and Ahmed, 2009; Dias et al., 2018; Hashimoto et al., 2018). By contrast, CD4^+^ T cells were shown to be activated during the chronic phase of CHIKV infection, playing a primary role in the pathogenesis of CHIKV-induced joint inflammation (Hoarau et al., 2010; Teo et al., 2013). An elegant study by Teo et al. (2017) illustrated the essential role of CD4^+^ T cells in the CHIKV-induced joint inflammation, as transferring splenic CD4^+^ T cells from CHIKV-infected wild-type mouse into CHIKV-infected T cell receptor-deficient (Tcr^−/−^) mice recapitulated severe joint disease (Teo et al., 2017).

The complex interactions between CHIKV and the host can determine the outcome of the viral infection. Although co-evolution of the virus with the host has led to the development of specialised immune responses to control viral replication, CHIKV, like many other pathogens, has evolved to evade the immune response of the host by hijacking antiviral pathways. For the development of therapeutic approaches aimed at enhancing early viral clearance and limiting the development of chronic disease, a greater understanding is required of both the acute and chronic phases of CHIKV disease and the role of host defence in the pathobiology of the disease.

## Antiviral strategies targeting the virus replication cycle

**Fig. 3.**
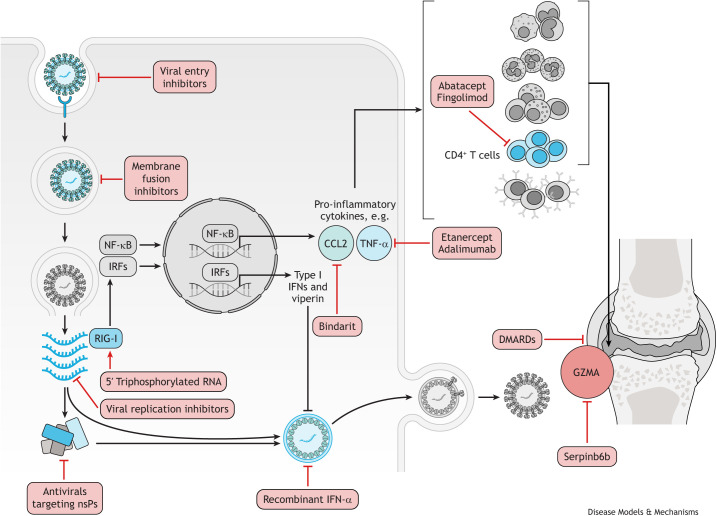
**CHIKV treatments targeting the viral life cycle, host defence mechanisms and host immunopathology.** Antivirals that block viral entry into the host cell include the natural compounds epigallocatechin gallate (Weber et al., 2015), flavaglines (Wintachai et al., 2015) and curcumin (Mounce et al., 2017)**.** *In vitro*, chloroquine was shown to increase the endosomal pH, which then prevents the fusion of CHIKV E1 glycoprotein with the endosomal membrane (Khan et al., 2010). Broad-spectrum antivirals, including ribavirin and 6-azauridine, inhibit viral replication by introducing mutations in the viral genome, whereas favipravir targets RNA polymerase and prevents viral replication (Briolant et al., 2004). The viral nsPs are also a potential target for the development of antiviral molecules, and several compounds have been identified to target different nsPs, including harringtonine (Bassetto et al., 2013; Das et al., 2016). In response to CHIKV infection, the host elicits a strong antiviral type I IFN; therefore, recombinant IFN-α has shown the ability to inhibit CHIKV replication *in vitro* (Briolant et al., 2004). Furthermore, activating the RIG-I receptor with 5′ triphosphorylated RNA can augment this host response (Olagnier et al., 2014). A prolonged pro-inflammatory host response to CHIKV infection can lead to the chronic disease; therefore, some treatments are aimed at dampening this response. Inhibiting the chemokine CCL2 with bindarit ameliorated the inflammation in joints and skeletal muscles in mice (Rulli et al., 2011). TNF-α inhibitors, such as etanercept and adamimumab, were shown to reduce arthritic symptoms in patients (Blettery et al., 2016). CD4^+^ T cells also have a primary role in the pathogenesis of CHIKV-induced joint inflammation (Teo et al., 2017). Fingolimod efficiently suppressed CHIKV-induced joint pathology in virus-infected mice by blocking T-cell migration from the lymph nodes to the joints (Teo et al., 2017), and abatacept, which inhibits CD4^+^ T-cell priming in combination with neutralising anti-CHIKV monoclonal antibody, reduced T-cell accumulation in the joints (Miner et al., 2017). This resulted in ameliorated joint inflammation and decrease in proinflammatory cytokine secretion (Miner et al., 2017). The pro-inflammatory granule GZMA is released by natural killer cells and CD8^+^ T cells in response to CHIKV infection, and the GZMA inhibitor, Serpinb6b, was shown to reduce joint inflammation (Wilson et al., 2017). DMARDs, including hydroxychloroquine, sulfasalazine and methotrexate, have been trialled for chronic CHIKV arthritis (Ganu and Ganu, 2011; Martí-Carvajal et al., 2017; Ravindran and Alias, 2017). However, owing to contradictory results, it remains unclear whether the use of specific DMARDs is effective in treating chronic CHIKV arthritis. CHIKV, chikungunya virus; DMARD, disease-modifying antirheumatic drug; GZMA, granzyme A; IFN, interferon; IRF, interferon regulatory factor; NF-κB, nuclear factor kappa B; nsP, non-structural protein; RIG-I, retinoic acid-inducible gene I; TNF-α, tumour necrosis factor alpha.

Viruses are obligatory intracellular microbes that require the host cell to replicate; therefore, it is difficult to define virus-specific functions as suitable targets for antiviral therapy. Nevertheless, extensive research has identified several molecules with anti-CHIKV potential, targeting the virus and/or the host. This section will summarise the antiviral strategies directed at the pathogen itself, as well as host cell pathways or molecules important for viral replication (Fig. 3). Currently, no approved antiviral treatment is available for CHIKV infection.

### Antiviral strategies targeting the virus

One approach for identifying novel anti-CHIKV therapies is to assess compounds that have shown antiviral potential against other viruses. Known compounds tested against CHIKV that have shown the potential to block viral entry *in vitro* include chloroquine, arbidol and derivatives (Di Mola et al., 2014), epigallocatechin gallate (EGCG; Weber et al., 2015), flavaglines (Wintachai et al., 2015), curcumin (Mounce et al., 2017) and phenothiazines (Pohjala et al., 2011). Chloroquine is an anti-malarial drug with anti-inflammatory properties, which is also used to treat a variety of inflammatory diseases, including RA, osteoarthritis and lupus. In *in vitro* studies, chloroquine showed direct antiviral activity against flaviviruses, retroviruses and coronaviruses (Savarino et al., 2003). Chloroquine treatment of CHIKV-infected Vero cells strongly inhibited viral replication, and the underlying mechanism is thought to be associated with an increase in endosomal pH that prevents the fusion of E1 glycoprotein with the endosomal membrane (Khan et al., 2010). However, in a clinical trial in Réunion Island, oral chloroquine had no significant effect on viremia or duration of symptoms compared to placebo treatment (De Lamballerie et al., 2008). A comparable study performed in India showed that chloroquine improved patient symptoms but failed to offer any advantage over treatment with the NSAID meloxicam (Chopra et al., 2014). Furthermore, another clinical trial and an NHP study suggested that chloroquine had a negative impact on the immunological response, causing a possible delay in viral clearance (Roques et al., 2018). The events leading to the revocation of chloroquine for COVID-19 treatment, owing to its inefficacy in hospitalised patients, further highlight the importance of translating *in vitro* studies to carefully planned animal studies and/or clinical trials.

Natural compounds mostly derived from plants, roots and spices have also been investigated for their anti-CHIKV activity. Among the first compounds to be tested *in vitro* against CHIKV was EGCG, the major constituent of green tea. EGCG was shown to prevent CHIKV entry into HEK293T cells, inhibiting its replication (Weber et al., 2015). Other natural compounds that have shown the ability to block CHIKV entry *in vitro* include flavaglines (Wintachai et al., 2012) and curcumin (von Rhein et al., 2016). Flavaglines are known ligands of prohibitin, and, recently, prohibitin-1 has been identified as a potential cellular receptor used by CHIKV to enter mammalian cells (Wintachai et al., 2012). Flavaglines were shown to inhibit CHIKV replication in HEK293T cells only when compounds were added prior to infection, which indicates that flavaglines interfere with CHIKV entry. This observation was further confirmed by the significant reduction in the colocalisation of CHIKV E2 protein with prohibitin-1 when incubated in the presence of flavaglines (Wintachai et al., 2015).

Other antiviral compounds have been shown to inhibit CHIKV replication, including well-known and characterised broad-spectrum antiviral agents, such as favipiravir (Delang et al., 2014), ribavirin, 6-azauridine, and glycyrrhizin. A study by Briolant et al. (2004) evaluated the anti-CHIKV activity and cytotoxicity of ribavirin, 6-azauridine and glycyrrhizin in Vero cells. 6-Azauridine was shown to reduce virus-induced cytopathogenicity and was more potent than ribavirin, inhibiting CHIKV replication at very-low concentrations (S et al., 2004). Glycyrrhizin inhibited CHIKV in Vero cells; however, this was only achieved at high subtoxic concentrations (Briolant et al., 2004). Ribavirin showed potent antiviral activity against CHIKV and exhibited synergistic effects with doxycycline (Rothan et al., 2015) and with recombinant IFN-α2 *in vitro* (Briolant et al., 2004). Despite the potent antiviral properties of ribavirin *in vitro*, this did not translate *in vivo*. A small non-randomised clinical trial (*n*=20) showed no evidence of greater efficacy of ribavirin compared to placebo (Ravichandran and Manian, 2008).

The replication cycle of CHIKV can also be utilised to identify novel targets for the development of antiviral compounds. The four non-structural proteins (nsPs) represent interesting targets for the identification of selective CHIKV inhibitors, as these proteins possess enzymatic activities that are essential for virus replication. An *in silico* study by Das et al. (2016) designed compounds predicted to interact with the CHIKV active centre of nsP2 proteases. Several of these identified compounds were then shown to inhibit nsP2 functionality, as well as suppress CHIKV replication in BHK-21 cells (Das et al., 2016). Bassetto et al. (2013) performed a virtual screening simulation of more than 5 million compounds on the CHIKV nsP2 protease. A series of 26 compounds was identified after performing analysis of the structure–activity relationship (Box 1). From those, only nine compounds were tested *in vitro* and were shown to inhibit CHIKV replication in Vero cells at non-cytotoxic concentrations (Bassetto et al., 2013).

In recent years, the in-depth characterisation of CHIKV nsP3 functional domains has identified nsP3 as a potential drug target. The N-terminal macrodomain of nsP3 is highly conserved amongst alphaviruses and other positive-strand RNA viruses (Eckei et al., 2017). Therefore, the nsP3 macrodomain represents an ideal site for the development of antivirals with broad antiviral activity. *In silico* screenings have helped identify putative CHIKV nsP3 inhibitors that target specific binding sites in the macrodomain. *In vitro* analysis demonstrated that two of the selected molecules inhibited CHIKV replication in Huh-7 cells (Shimizu et al., 2020). This study highlights the importance of validating *in vitro* the antiviral properties and mode of action of inhibitors discovered using *in silico* methods.

Other CHIKV proteins have also been identified as potential targets for the development of inhibitors. The benzimidazole-related compound, designated Compound A, was found to inhibit several CHIKV strains at very-low concentrations. Compound A was identified as an inhibitor of CHIKV RNA polymerase by it targeting a residue in the nsP4 protein and therefore inhibiting the replication of CHIKV in Vero cells (Wada et al., 2017). Kaur et al. (2013), used an immunofluorescence-based screening platform to identify 44 compounds that resulted in >70% inhibition of CHIKV replication. Among these, harringtonine, hypocrellin A, rottlerin and daunorubicin inhibited CHIKV replication in BHK21 cells in a dose-dependent manner, and harringtonine was identified as the most potent antiviral compound. Harringtonine treatment of CHIKV-infected cells resulted in a decrease in CHIKV RNA products and synthesis of nsP3 and E2 proteins, which suggested that harringtonine interfered with viral protein translation (Kaur et al., 2013).

Several promising antiviral compounds have been developed; however, most of them still have to be validated *in vivo* and in clinical trials. The use of re-purposed antiviral compounds, such as chloroquine, ribavirin and favipiravir, for the treatment of CHIKV has its advantages as these have already been intensively evaluated in patients, potentially fast-tracking clinical trials for them against CHIKV. So far, however, these have failed to maintain their efficacy *in vivo* and in clinical trials (e.g. chloroquine and ribavirin). Although targeting the virus provides a specific and direct inhibitory effect, there is the risk of the virus mutating and developing resistance to antiviral therapy (Delang et al., 2014). An alternative antiviral approach, with the potential to lower the likelihood of drug resistance, is the identification of host pathways as possible targets for CHIKV inhibitors.

### Antiviral strategies targeting host defence mechanisms

In addition to targeting the pathogen itself, novel antiviral approaches target host cell pathways or molecules to interfere with CHIKV replication, an approach that has shown considerable efficacy with other viral pathogens. A genome loss-of-function screen in which HEK293 cells were transfected with a library of short interfering RNAs (siRNAs) targeting host cell genes prior to CHIKV infection identified a diverse range of novel host factors and pathways, including ATPases, calmodulin signalling and fatty acid synthesis, which were essential for CHIKV replication (pro-viral) or limited its replication (antiviral) (Karlas et al., 2016). Two compounds, the calmodulin inhibitor pimozide and the fatty acid synthesis inhibitor TOFA, demonstrated potent antiviral effects in HeLa and HEK-293T cells and C57BL/6 mice. These effects were enhanced when the drugs were used in combination, suggesting that the use of drugs targeting different cellular pathways may enhance antiviral effects and reduce the chance of CHIKV developing resistance (Karlas et al., 2016). Another approach includes targeting host cell receptors, such as intracellular RIG-I receptors that recognise dsRNA and activate multiple antiviral factors blocking viral replication. Experimentally, an optimised RIG-I agonist has been shown to trigger RIG-I and protect lung fibroblast MRC-5 cells from CHIKV infection (Olagnier et al., 2014), demonstrating a promising approach for therapeutic development.

Targeting host factors can offer several advantages over just targeting the virus itself. For instance, modulating host pathways may allow the inhibition of all viruses that depend on that particular function. Additionally, combining these compounds with specific direct antiviral compounds may lead to synergistic effects with the potential to develop more comprehensive treatment plans that target multiple pathways. Chatterjee et al. (2022) recently identified checkpoint kinase proteins Chk1 and Chk2 (also known as CHEK1 and CHEK2), which are commonly known as DNA damage response proteins, as host factors that are involved in CHIKV replication. Similarly, De Caluwé et al. (2021) characterised the role of the CD147 protein complex in CHIKV entry into host cells. With inhibitors for both of these host targets currently being used to treat other disorders, establishing whether these compounds suppress viral replication merits experimental testing. The number of novel therapeutic compounds aimed at inhibiting host molecules involved in CHIKV replication is steadily growing. However, as these approaches target host factors, potential serious adverse or off-target effects may occur.

Thus far, several classes of compounds have shown the potential to inhibit CHIKV either by directly targeting the virus or by targeting host cell factors; however, many of these inhibitors have only been tested *in vitro*. Currently, there is no standardised protocol to determine the efficacy and toxicity of antiviral drugs *in vitro*, which makes comparison of the different hits extremely challenging. The efficacy and toxicity values vary widely depending on the cell line, assay method and viral strain (Battisti et al., 2021). A more comprehensive validation of potential antiviral drugs *in vitro* could help make translational research more robust and efficient (Emmerich et al., 2021). The use of animal models to further examine the potential of these antivirals is clearly needed, which will help to determine their effect in different stages of CHIKV disease and enable clinical translation.

## Therapeutic targets to limit the development of CHIKV-associated immunopathology

The recent outbreaks of chikungunya have allowed for a better understanding of the innate and adaptive immune responses induced by CHIKV infection (reviewed in the ‘CHIKV infection and host immune responses’ section). Many responses that contribute to CHIKV immunopathology are also required for protection against viral infections. Therefore, an important consideration for the development of new therapeutic interventions is to target excessive inflammation without compromising antiviral immunity. In this section, we will discuss new potential therapeutic avenues aimed at enhancing early viral clearance and limiting the development of chronic disease (Fig. 3).

IFNs play an essential role in the control of CHIKV infection and provide a promising avenue for the development of antivirals against this virus. One of the most well characterised and effective clinical applications for IFNs is the use of recombinant IFN-α against chronic hepatitis C virus (Hoofnagle and Seeff, 2006). The use of exogenous IFNs was also investigated for CHIKV infection, as Briolant et al. (2004) demonstrated that treatment with recombinant IFN-α inhibited CHIKV replication in Vero cells. Furthermore, it has been shown that a combination approach of IFN-α paired with the antiviral drug ribavirin resulted in a synergistic inhibitory effect on CHIKV replication *in vitro* (Gallegos et al., 2016). Conversely, in addition to inhibiting viral replication, type I IFNs can promote arthritis. For instance, injection of polyinosinic acid–polycytidylic acid (poly I:C), which structurally resembles dsRNA and is a potent stimulator of type I IFN, into the feet of mice can induce arthritis (Prow et al., 2017). This indicates that further translational studies are required to determine the efficacy of this regimen, in order to find balance between antiviral activity and the inflammatory response.

The identification of appropriate pro-inflammatory mediators that can be targeted without compromising the protective antiviral responses has been a major objective in the development of novel therapeutics for CHIKV disease (Long and Heise, 2015). TNF-α is induced during CHIKV infection (Teng et al., 2015; Thanapati et al., 2017; Wilson et al., 2017), suggesting that TNF-α inhibitors could treat viral arthritis. In fact, treatment of patients with CHIKV with TNF-α antagonists, etanercept and adalimumab, showed promise with good tolerance without the reappearance of the viral infections manifestations (Blettery et al., 2016; Brunier et al., 2016). However, TNF-α also plays an important antiviral role (Rahman and McFadden, 2006). For example, treatment with etanercept resulted in a dramatic disease exacerbation and 100% mortality of mice infected with RRV, which closely resembles CHIKV (Zaid et al., 2011). The protective role of TNF-α in viral infections raises concerns about TNF antagonists for the treatment of patients with CHIKV during the acute phase. However, for patients with chronic manifestations of CHIKV, treatment with TNF-α antagonists has not been associated with obvious disease worsening (Guaraldo et al., 2018), and TNF-α antagonist treatment is included in guidelines to treat CHIKV chronic disease (Simon et al., 2015; Marques et al., 2017). A critical observation is the likelihood of compromising antiviral immunity in locations in which other arboviruses circulate. Ideally, therapies that target CHIKV immunopathology do not compromise the patient's ability to generate immunity in subsequent viral infections.

Another potential anti-inflammatory intervention is supressing CCL2 expression, which is strongly induced during CHIKV infection (Teng et al., 2015; Michlmayr et al., 2018), using bindarit. The treatment of CHIKV-infected mice with bindarit resulted in reduced monocyte recruitment, joint and skeletal muscle tissue swelling, and bone loss (Rulli et al., 2011). This suggests that bindarit, and other CCL2 inhibitors, could potentially be used as a treatment for CHIKV-induced arthritis in humans. However, in the absence of monocytes and macrophages, neutrophils and eosinophils are instead recruited into the joints of mice deficient for the CCL2 receptor, CCR2, promoting joint destruction (Poo et al., 2014a). This raises concerns in using anti-inflammatory interventions due to the potential risk of inadvertently promoting immunopathology.

GZMA was also shown to be elevated in the serum of patients with CHIKV, and its role in driving arthritic inflammation was demonstrated in infected mice (Schanoski et al., 2019). CHIKV-infected wild-type mice were treated with Serpinb6b, a murine GZMA inhibitor, which reduced arthritic inflammation, without compromising the antiviral activity against CHIKV (Wilson et al., 2017). Targeting and inhibiting the activity of GZMA may, therefore, be a potential therapeutic approach for CHIKV-induced arthritis. However, there are distinct differences between murine GZMA and human GZMA (Kaiserman et al., 2006; 2014), indicating the need for more refined translational studies.

Targeting pathogenic CD4^+^ T cells has also shown some promising results in animal models (Teo et al., 2017). For example, the sphingosine 1-phosphate receptor agonist, fingolimod, efficiently suppressed CHIKV-induced joint pathology in virus-infected mice by blocking CD4^+^ T-cell migration from lymphoid organs into the joints (Teo et al., 2017). Fingolimod is a clinically approved treatment for multiple sclerosis, although currently there are no clinical trials to assess efficacy in chronic CHIKV disease. Another approach has involved the use of biological disease-modifying antirheumatic drugs (DMARDs) such as abacept, which blocks CD4^+^ T cell co-stimulation (CTLA4-Ig), in combination with neutralising anti-CHIKV human monoclonal antibodies (mAbs). In CHIKV-infected mice, the combination therapy successfully reduced CD4^+^ T-cell accumulation in the joints and decreased proinflammatory cytokines, which ameliorated joint inflammation (Miner et al., 2017).

Owing to the fact that chronic CHIKV-induced arthritis resembles RA, the use of DMARDs, including hydroxychloroquine (HCQ), sulfasalazine and methotrexate (MTX) have been trialled for chronic CHIKV arthritis (Ganu and Ganu, 2011; Martí-Carvajal et al., 2017; Ravindran and Alias, 2017). A randomised controlled trial in patients with chronic CHIKV-induced arthritis compared the efficacy of combining MTX, sulfasalazine and HCQ versus HCQ monotherapy and showed that the combination of DMARDs was more effective than HCQ monotherapy (Ravindran and Alias, 2017). However, a longitudinal study showed that most patients displayed progression of disease even with continued treatment with these DMARDs (Bouquillard and Combe, 2009). Owing to conflicting results and the lack of statistical power in trials, it remains unclear whether the use of specific DMARDs is effective in treating chronic CHIKV arthritis.

Although the drugs discussed herein are an exciting avenue for targeting specific immunoinflammatory pathways, the high costs of these drugs might prevent their widespread use, especially in resource-poor settings. In addition, human data for the use of these therapies are very limited, inconclusive and, in most cases, non-existent.

## Therapeutic antibodies

Passive antibody therapy that involves the administration of convalescent serum to susceptible individuals to prevent or treat viral infections, such as measles (Park and Freeman, 1926) and influenza (Luke et al., 2010), has a long history and has recently been investigated for the treatment of severe acute respiratory syndrome coronavirus 2 (SARS-CoV-2) (Montelongo-Jauregui et al., 2020). Similarly, antibody therapy has been considered for treatment of alphaviruses, in which immune sera protected mice against pathology associated with Sindbis (Wust et al., 1989), Semliki Forest (Boere et al., 1983) and Venezuelan equine encephalitis viruses (Rabinowitz and Adler, 1973). These studies encouraged the exploration of antibody therapy to combat CHIKV infection.

An elegant study by Couderc et al. (2008) showed that the passive transfer of anti-CHIKV human polyclonal antibodies from donors in the convalescent phase of CHIKV disease prevented and treated CHIKV in adult and neonate mice, providing the rationale that isolation and administration of CHIKV immunoglobulins may be a safe and effective strategy for CHIKV prophylaxis and/or treatment. Building upon this, studies have investigated the use of mAbs to target specific CHIKV antigens, such as E1 and E2 glycoproteins that block viral attachment and entry into host cells (Bréhin et al., 2008; Selvarajah et al., 2013; Masrinoul et al., 2014; Smith et al., 2015; Weger-Lucarelli et al., 2015; Clayton, 2016). Human mAbs isolated from recovered patients have effectively inhibited CHIKV infection *in vitro* and *in vivo* (Selvarajah et al., 2013; Smith et al., 2015)*.* For instance, the human mAb C9, which recognises the acid-sensitive region in CHIKV E2 that is critical for viral fusion and entry into host cells, provided complete protection against CHIKV viremia and CHIKV-induced arthritis when administered prophylactically to CHIKV-infected mice. In addition, when mAb C9 was administered therapeutically to mice at 8 h or 18 h after a lethal dose of CHIKV, all mice were protected against lethality (Selvarajah et al., 2013). This was also demonstrated in immunocompromised Ifnar^−/−^ mice, which were protected from a lethal CHIKV challenge even 60 h post-infection when treated with human mAbs that target the A domain of E2, known to be required for viral fusion (Smith et al., 2015). A study by Pal et al. (2013) screened 230 mouse anti-CHIKV mAbs and identified four mAbs that provided complete protection when administered prophylactically to Ifnar^−/−^ mice (Pal et al., 2013). Interestingly, in humans, it has been demonstrated that anti-CHIKV antibodies have wide-ranging neutralising activity against viruses belonging to different CHIKV genotypes (Smith et al., 2015), and antibodies arising from infection with a closely related non-CHIKV alphavirus could have cross-neutralising activity against CHIKV (Kam et al., 2015).

Despite the therapeutic potential of anti-CHIKV antibodies, it is important to consider that viruses can develop resistance to neutralising antibodies by undergoing genetic mutations to inhibit antigen specificity and antigen–antibody binding (Lee et al., 2011), or through indirect immune evasion strategies, including cell-to-cell transmission, where the virus can directly be transmitted between cells, bypassing the extracellular space (Hahon and Zimmerman, 1970; Lee et al., 2011; reviewed in Jin and Simmons, 2019). A number of neutralisation-escape mutants have been identified for CHIKV, and *in vitro* studies reported that these can be generated under the selective pressure of mAbs (Lee et al., 2011). Combining neutralising mAbs that target distinct viral epitopes can prevent virus immune escape. Pal et al. (2013) demonstrated that the combination of pairs of mAbs protected mice from lethal doses of CHIKV and extended the treatment window. The most promising pair of mAbs targeted distinct epitopes and was shown to limit the generation of viral resistance, possibly by blocking multiple stages of viral entry. The efficacy of this combination of mAbs was further evaluated in an NHP model of CHIKV infection. The treatment of rhesus macaques with these mAbs reduced viral spread and infection; however, viral RNA persisted in the presence of mAb therapy, which could reflect populations of cells that actively replicate viral RNA (Pal et al., 2014). Therefore, the use of mAbs against CHIKV infection can provide immediate immune-mediated viral suppression, although high viral loads and viral RNA persistence observed in CHIKV disease complicate the use of mAb therapy.

The practical use of antibody-based therapies for viral infections poses several limitations. For example, antibody therapy works best if administered early during the acute phase of the infection when viral loads are high. The administration of exogenous antibodies once an infection is established has little benefit. In addition, the development of antibody-based therapies is expensive. As infectious disease incidences are higher in low-income countries, the adoption of these complex and costly antibody-based therapies would be challenging.

## Vaccine development

Many tropical diseases are often neglected as they disproportionally affect low-income populations and do not always, therefore, provide the investment potential that companies developing vaccine candidates demand (Rezza, 2015; Rezza and Weaver, 2019). To date, no vaccine has been licensed to control CHIKV infections. However, CHIKV vaccine development started in the 1960s, soon after the virus was first isolated, and CHIKV vaccine candidates that balance both immunogenicity and safety have been developed and tested in clinical trials. CHIKV has limited antigenic diversity, and, after infection, the production of anti-CHIKV neutralising antibodies is typically enough to protect against re-infection (Kam et al., 2012); therefore, vaccine development continues to be an attractive approach to control urban CHIKV outbreaks.

**Fig. 4.**
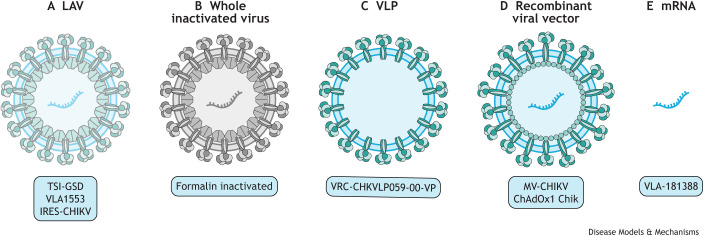
**Several platforms used for the development of CHIKV vaccines that have entered human clinical trial testing.** (A) LAV vaccines are derived from CHIKV, with structural changes introduced to reduce its virulence but retain its immunogenicity. The CHIKV LAV vaccines that have reached human clinical trials include TSI-GSD (The Salk Institute-Government Services Division), which showed promising results in phase I and II trials, but, owing to limited funding and uncertainty about safety, the development of this vaccine was terminated (Edelman et al., 2000); the Δ5nsP3 vaccine (VLA1553), which recently entered phase III clinical trial (NCT04546724); and IRES-CHIKV, which is in its late preclinical stages, in which immunogenicity and efficacy have been tested in mice and non-human primates (Plante et al., 2011; 2015). (B) The use of whole inactivated virus via chemical treatment, with formalin, was one of the first strategies for the development of a vaccine for CHIKV. The formalin-inactivated CHIKV vaccine entered clinical phase I (Harrison et al., 1971); however, despite the good safety, tolerability and immunogenicity profiles, the vaccine programme was discontinued. (C) VLP vaccines closely resemble the wild-type virus by containing self-assembled structural proteins but no viral genetic material. A CHIKV VLP vaccine candidate, VRC-CHKVLP059-00-VP, has now advanced into clinical phase II evaluation (NCT02562482) (Akahata et al., 2010). (D) Chimeric or recombinant viral-vectored vaccines are obtained by incorporating genetic elements of CHIKV into a vector virus genome. The recombinant viral-vectored vaccine MV-CHIKV, which uses attenuated measles virus as a vector, is currently in clinical phase II (NCT02861586), and ChAdOx1 Chik vaccine, known as the chimpanzee adenovirus Oxford 1 as it uses the Simian adenovirus as a vector, is in phase I of a clinical trial (NCT03590392). (E) mRNA vaccines are the newest strategy for the development of CHIKV vaccine candidates, based on the delivery of engineered mRNAs that can instruct host cells to express viral antigens, mimicking a viral infection that could elicit an immune response and lead to the generation of antibodies. There is currently one CHIKV mRNA vaccine, VLA-181388, in phase I clinical trial (NCT03325075) (Goyal et al., 2018). CHIKV, chikungunya virus; LAV, live-attenuated virus; VLP, virus-like particle.

Various strategies have been adopted to develop vaccines and these can be classified as live-attenuated virus (LAV) vaccines, whole inactivated viral vaccines, subunit or virus-like particle (VLP) vaccines, chimeric or recombinant viral-vectored vaccines, and mRNA vaccines. This Review focuses on the recent progress made in vaccine development, in particular, vaccines that have advanced into clinical trials (Table 1 and Fig. 4). Of note, there are currently no whole inactivated vaccines that are being tested in clinical trials for CHIKV, despite early work on a formalin-inactivated CHIKV vaccine that displayed good safety, tolerability and immunogenicity profiles in a phase I clinical trial (Harrison et al., 1971).

**Table 1.**
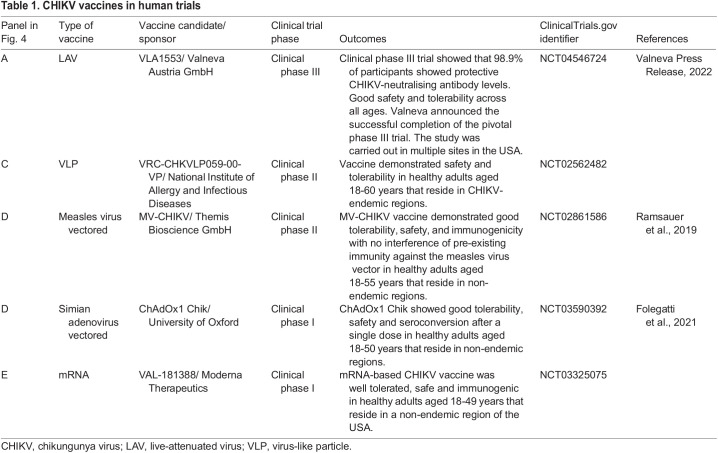
CHIKV vaccines in human trials

LAVs are derived from CHIKV, with structural changes introduced to reduce its virulence but retain its immunogenicity. LAV vaccine candidates contain very-specific mutations or alterations of the original virus genome that increase specificity, improve safety and offer high immunogenicity, providing protection with one single dose of the vaccine. Early immune responses to CHIKV are activated by viral nsPs; therefore, several LAV vaccines have been designed to target nsPs. A recent paper on the LAV vaccine Δ5nsP3, in which the replicase region of nsP3 was deleted, showed that this vaccine generated a strong, long-lasting and protective antibody response upon a single immunisation in *Cynomolgus macaques*. This vaccine also generated significant IFN-γ T-cell responses and activation of specific CD4^+^ T cells, which are required for B-cell maturation, and long-lived B cells that correlate with a long-lasting protective effects (Roques et al., 2017). Of note, the Δ5nsP3 vaccine candidate named VLA1553 has recently advanced to clinical phase III trials, having previously shown good immunogenicity and safety profiles in healthy adults (NCT04546724). Several other strategies to attenuate CHIKV have been exploited for vaccine development, such as complete capsid deletion (Zhang et al., 2019), the introduction of point mutations in nsPs (Chan et al., 2019), and the replacement of the subgenomic promoter in a cDNA CHIKV clone by the internal ribosome entry site (IRES) from encephalomyocarditis virus (Plante et al., 2011). Owing to its high immunogenic profile, LAV is the platform with one of the best prospects.

Another successful vaccine platform is the VLP vaccines that closely resemble the wild-type virus by containing self-assembled structural proteins but no viral genetic material. VLPs are highly immunogenic, typically elicit high-titre neutralising antibodies, and are considered to be safe as they are non-replicative, non-infectious viral constructs (Akahata et al., 2010; DeZure et al., 2016). A CHIKV VLP candidate vaccine, VRC-CHKVLP059-00-VP, has now advanced into clinical phase II evaluation (NCT02562482) following reassuring preclinical and early clinical evaluation (Akahata et al., 2010). This vaccine is produced in mammalian cells (HEK293 cells) by transfecting expression vectors encoding CHIKV structural proteins (C-E3-E2-6K-E1) from a West African CHIKV strain. This results in the production of VLPs that mimic wild-type virus with E1/E2 glycoproteins. Immunisation of NHPs with VLPs resulted in high neutralising antibody titres, which protected the adult rhesus macaques against viremia after high-dose challenge (Akahata et al., 2010). A potential drawback of this vaccine platform is that VLPs require multiple dosages to induce maximal immunogenicity, which in turn can increase the cost of manufacturing. Additionally, phase I and II clinical trials are carried out in adults between the ages of 18 and 60 years of age, which introduces a knowledge gap regarding the CHIKV VLP vaccine efficacy in the elderly. A study by Arévalo et al. (2019) combined CHIKV VLP vaccine with different adjuvants to enhance immunogenicity and protection in adult and aged mice. Results showed that VLP vaccine alone was able to protect adult mice against CHIKV disease; however, the disease was exacerbated in aged mice when vaccinated with VLP alone or with an adjuvant. This study suggests the need for an improved or alternative vaccine approach to protect the elderly against CHIKV infections (Arévalo et al., 2019).

Chimeric or recombinant viral-vectored vaccines are obtained by incorporating genetic elements of CHIKV into a vector virus genome. Several different virus vectors have been used for CHIKV vaccine development and include measles virus, alphavirus, vaccinia virus, adenovirus and vesiculovirus. Recombinant viral-vectored vaccines are live replicating viruses with avirulent vectors, which make these vaccines safe and highly immunogenic. The most developed of these vaccines for CHIKV is based on the measles virus vector (MV). The MV-CHIKV vaccine uses attenuated measles virus, which contains a large structural coding gene (C-E3-E2-6K-E1) derived from the East/Central/South African (ECSA) CHIKV strain. MV-CHIKV vaccine has performed well in clinical phases I and II, demonstrating good tolerability, safety and immunogenicity with no interference of pre-existing immunity against the MV (Ramsauer et al., 2015; 2019). NHP models can further demonstrate vaccine efficacy before proceeding into pivotal phase III clinical trials, as they closely mimic human CHIKV infection responses. Studies have revealed that cynomolgus macaques vaccinated with MV-CHIKV demonstrated a robust immune response and protection from any clinical symptoms of the disease and viremia after challenge with wild-type CHIKV (Rossi et al., 2019). However, important aspects, such as the contribution of T cells and the length of the immune responses, were not addressed in this study and are critical for the progression of this vaccine. Nevertheless, MV-CHIKV is a promising vaccine candidate for CHIKV.

The newest category of vaccine to advance into clinical trials is mRNA vaccine, currently used for SARS-CoV-2 (Jackson et al., 2020). One approach for using this platform is based on the delivery of engineered mRNAs that can instruct host cells to express viral antigens, mimicking a viral infection that could elicit an immune response and lead to the generation of antibodies. Moderna Therapeutics have developed an mRNA vaccine candidate for CHIKV, VAL-181388, that is currently in a phase I clinical trial (NCT03325075). No preclinical data have been published, but it has been reported that a single dose of CHIKV mRNA vaccine protected mice from infection and generated a robust antibody response in NHPs (Goyal et al., 2018). The recent development of COVID-19 vaccines may, hopefully, instigate a new era for the development and approval of vaccines for CHIKV and other neglected infectious diseases that disproportionally affect low-income regions.

Knowledge of the lineage-specific variations (Box 3) in virulence and cross-protection among CHIKV strains is an important consideration when developing disease prevention therapies, such as vaccines, and other treatments. A study by Langsjoen et al. (2018) showed that in type I IFN receptor knockout mice, distinct CHIKV lineages differ in their virulence. West African (WA) strains produced higher levels of viremia than ECSA, Indian Ocean lineage (IOL) and Asian/American strains, whereas the Asian/American strains, in general, appeared to be more attenuated than WA, ECSA and Asian strains. In addition, the IOL-derived, LAV strain, IRES-CHIKV, provided protection in mice and NHPs against the Asian/American strain and WA strain, suggesting that the IOL-based vaccine offers cross-protection against strains from other CHIKV lineages (Langsjoen et al., 2018). This is in accordance with the general opinion that CHIKV has a single serotype (Volk et al., 2010; Chua et al., 2016; Langsjoen et al., 2018). Nevertheless, further research is necessary to correlate the observed variation of CHIKV virulence with pathogenicity and vaccine efficacy.

Vaccine efficacy is traditionally demonstrated in randomised, controlled vaccine phase III trials in an outbreak area (Marshall and Baylor, 2011). However, it is extremely difficult to predict when and where CHIKV outbreaks will happen due to the sporadic epidemiology of this disease (Rezza and Weaver, 2019). The current vaccine candidate in clinical trial III, VLA1553, relied on neutralising antibody titres as the endpoints (NCT04546724); however, this surrogate of protection is still controversial (Yang et al., 2017). Completion of phase III vaccine efficacy trials depends not just on a CHIKV outbreak happening somewhere, but in a specific population that can be enrolled and followed during a longitudinal trial. The ongoing outbreaks of CHIKV worldwide (Bettis et al., 2022) provide some opportunity for carrying out these efficacy trials; however, thus far, vaccine candidates have not been able to leverage these outbreaks for phase III efficacy trials that are required for vaccines to be licensed.

## Future perspectives

The spread of CHIKV across the globe confirms that the virus is expanding at a significant rate and has the potential to migrate to new geographic areas. Millions of people in the tropics and subtropics have already been affected or are at high risk of infection, and countries with mild climates may experience severe outbreaks due to the year-round presence of the mosquito vector *A. albopictus*. Rapid global warming is thought to have long-term implications for the prevention and control of vector-borne diseases, as vectors thrive in warmer conditions (Brady et al., 2013). Climate change is predicted to get worse in the near future, and mathematical models predict a geographical expansion of climates that are suitable for a number of vector-borne diseases, including CHIKV (Fischer et al., 2013; Tjaden et al., 2017; Carlson et al., 2022). In a warming world, there is also increased potential for pathogens and vectors to evolve due to higher replication rates (Fay et al., 2021). Such evolution could play an important part in vector-borne disease emergence, re-emergence and spread. The origin and the extent of a future CHIKV outbreak are difficult to foresee, which underlines the importance of developing effective countermeasures. Currently, no specific antiviral treatments or vaccines are available to prevent CHIKV infection, and the containment measures for future outbreaks mainly rely on the interruption of the transmission chain by controlling vector density and distribution (Rezza and Weaver, 2019).

Effective therapeutic and preventative approaches for CHIKV infection are urgently required. In the area of antiviral therapy, there are several compounds that have been shown to be effective at inhibiting CHIKV replication. However, owing to limited knowledge on CHIKV pathogenesis, viral mutation dynamics and a lack of studies performed in animal models and humans, this has proven insufficient for licencing an antiviral therapy for CHIKV disease.

Neutralising mAbs have proven to be effective against CHIKV infection, but additional research is required to understand the kinetics of CHIKV infection. This will in turn inform medical practitioners of optimum treatment timings, doses and whether mAbs can prevent and/or treat chronic CHIKV-induced arthritis.

Notably, there has been promising progress in the development of vaccine candidates for CHIKV disease. For example, the LAV, VLA1553, has now entered phase III clinical trial. However, licensing a vaccine for CHIKV is challenging due to the amount of funding required to bring a new vaccine to the market.

Despite this, the ongoing COVID-19 pandemic has challenged our paradigm of what is possible in vaccine development, proving that fast vaccine development can be achieved when there is a global effort and sufficient resources. However, what does this signify for the development of CHIKV and other vaccines? For a start, it may establish the use of mRNA vaccines, which until now had not been approved for general use in people. Currently, the CHIKV mRNA vaccine candidate, VAL-181388, is in phase I clinical trial (NCT03325075). This may facilitate efficacy and safety testing in humans, pushing vaccine development forward to the next phase of the clinical trials. However, for a vaccine to be developed at a comparable speed, the infection levels need to be high to enable rapid and large clinical trials, with enormous amounts of funding.

The development of vaccines for CHIKV and other neglected tropical viral diseases is not considered highly profitable by industry. For example, the global market for CHIKV vaccine has been estimated to be ∼US$500 million annually (Valneva Press Release, 2022), whereas the global influenza vaccine market value is estimated to reach US$7.547 billion by 2024 (Global Influenza Vaccine Market Report, 2018-2024). Strategies have been implemented to overcome this problem, including the creation of public and private partnerships, in particular the Coalition for Epidemic Preparedness Innovations (CEPI) (see CEPI ‘Priority diseases’ information), which are essential to incentivise and accelerate vaccine development against emerging infectious diseases (Rezza and Weaver, 2019). Currently, CEPI is providing funding worth US$14.1 million for the development of an inactivated whole virion vaccine against CHIKV (BioPharma Reporter, 2020).

Although important advances have been made in developing novel therapeutics for CHIKV disease, further characterisation of the molecular mechanisms employed by the virus, host–pathogen interactions and inflammatory responses that lead to disease progression are required in order to design better and more targeted approaches.
